# Improving public hospital efficiency and fiscal space implications: the case of Mauritius

**DOI:** 10.1186/s12939-020-01262-9

**Published:** 2020-09-04

**Authors:** Ajoy Nundoochan

**Affiliations:** World Health Organization Country Office, Port Louis, Mauritius

**Keywords:** Technical efficiency, Stochastic frontier analysis, Fiscal space

## Abstract

**Background:**

General Government Health Expenditure (GGHE) in Mauritius accounted for only 10% of General Government Expenditure for the fiscal year 2018. This is less than the pledge taken under the Abuja 2001 Declaration to allocate at least 15% of national budget to the health sector. The latest National Health Accounts also urged for an expansion in the fiscal space for health. As public hospitals in Mauritius absorb 70% of GGHE, maximising returns of hospitals is essential to achieve Universal Health Coverage. More so, as Mauritius is bracing for its worst recession in 40 years in the aftermath of the COVID-19 pandemic public health financing will be heavily impacted. A thorough assessment of hospital efficiency and its implications on effective public health financing and fiscal space creation is, therefore, vital to inform ongoing health reform agenda.

**Objectives:**

This paper aims to examine the trend in hospital technical efficiency over the period 2001–2017, to measure the elasticity of hospital output to changes in inputs variables and to assess the impact of improved hospital technical efficiency in terms of fiscal space creation.

**Methods:**

Annual health statistics released by the Ministry of Health and Wellness and national budget of the Ministry of Finance, Economic Planning and Development were the principal sources of data. Applying Stochastic Frontier Analysis, technical efficiency of public regional hospitals was estimated under Cobb–Douglas, Translog and Multi-output distance functions, using STATA 11. Hospital beds, doctors, nurses and non-medical staff were used as input variables. Output variable combined inpatients and outpatients seen at Accident Emergency, Sorted and Unsorted departments. Efficiency scores were used to determine potential efficiency savings and fiscal space creation.

**Findings:**

Mean technical efficiency scores, using the Cobb Douglas, Translog and Multi-output functions, were estimated at 0.83, 0.84 and 0.89, respectively. Nurses and beds are the most important factors in hospital production, as a 1% increase in the number of beds and nurses, result in an increase in hospital outputs by 0.73 and 0.51%, respectively. If hospitals are to increase their inputs by 1%, their outputs will increase by 1.16%. Hospital output process has an increasing return to scale. With technical efficiencies improving to scores of 0.95 and 1.0 in 2021–2022, potential savings and fiscal space creation at hospital level, would amount to MUR 633 million (US$ 16.2 million) and MUR 1161 million (US$ 29.6 million), respectively.

**Conclusion:**

Fiscal space creation through full technical efficiency, is estimated to represent 8.9 and 9.2% of GGHE in fiscal year 2021–2022 and 2022–2023, respectively. This will allow without any restrictions the funding of the national response for HIV, vaccine preventable diseases as well as building a resilient health system to mitigate impact of emerging infectious diseases as experienced with COVID-19.

## Background

Countries took a firm commitment to ensure Universal Health Coverage (UHC) with the endorsement of the 2030 agenda for Sustainable Development. The goal of UHC is that every individual and community, irrespective of their circumstances, receive the health services they need without risking financial hardship [1]. A core determinant influencing rapid progress towards UHC is efficient use of resources, along with adequate resource mobilisation. The World Health Report (2010) shed light on how to raise domestic funds for health, promote financial risk protection relating to health, and achieve efficiency in use of domestic resources allocated for health. The report posited that financial resources is not a sine qua non to achieve UHC as effective implementation of efficiency strategies is also essential. Indeed evidence concluded that 20 to 40% of resources spent on health are lost due to inefficiency. A wide array of common sources for inefficiency have been identified. These include inadequate controls on supply-chain agents, prescribers and dispensers; inadequate pharmaceutical regulatory structures/mechanisms; inappropriate hospital admissions and length of stay; asymmetric level of managerial resources for coordination and control with too many hospitals and inpatient beds in some areas and not enough in others; unclear resource allocation guidance; lack of transparency; poor accountability and governance mechanism; and inefficient mix for health interventions with resources skewed to curative services at the expenses of health promotion and prevention interventions [2, 3].

Several studies on assessment of national health systems efficiency focused on health outcomes and performance; including the World Health Report 2000 which ranked Mauritius health system performance eighty-fourth with a score of 0.691 [4]. A comparative efficiency analysis among 191 national health systems in 2001 concluded that, albeit rising per capita health expenditure is decisive for low income countries, significant gains in terms of health outcomes are doable using available resources efficiently [5]. In the same vein evidence demonstrated that countries with similar socio-economic profiles often have wide variation not only in their health outcomes, but also in their service coverage rates and degree of financial protection [6, 7]. Consequently, the discourse that a desirable condition to achieve UHC is adequate financial protection and health service coverage has shaped the focus of researchers to compare the performance of national health systems in making progress towards UHC goals. WHO recently assessed the performance of 83 low and middle-income countries towards achieving UHC. Evidence generated questioned the assertion that low and middle-income countries below a certain threshold of health expenditure cannot make progress towards UHC. However, put aside financial protection, health systems efficiency is key to move countries towards achieving UHC [8].

The concept of efficiency gathered much prominence when Farell (1957) building on earlier works of Debreu and Koopmans defined three types of efficiency [9–11]. A health care provider is technically efficient when it manages to produce the maximum outputs possible with the production factors or inputs put at their disposal. Besides the above referred output-oriented approach a provider is equally technical efficient when making use of minimal possible mix of production factors or inputs to produce a given output. The latter definition considers technical efficiency from an input-oriented perspective [12, 13].

On the other hand, allocative efficiency refers to an optimal combination of inputs deployed at a minimal cost to produce a given output amount. Any change in the mix of the inputs employed, while maintaining the same output amount, will entail additional cost implications [14]. Finally, economic efficiency is achieved only when both technical and allocative efficiency conditions are met. In this case the provider will employ the minimum level of inputs required coupled with that mix of inputs at minimal costs necessary to achieve that given output amount [15].

Distinct methods have been developed to estimate hospital efficiency. The most widely accepted objective measure of hospital efficiency is the production possibility frontier (PPF). PPF represents a locus of potentially efficient output combinations that an organization can employ at a point in time. The efficiency of a hospital is best demonstrated by the relationship between observed production and the PPF. Deviation of observed outputs from the PPF reflects the extent of technical inefficiency. Conversely, a hospital which does not produce on the PPF but below same is viewed as technically inefficient with the ratio of the actual to potential production measuring the scale of inefficiency.

Hospitals in significant number of countries absorb over half or even two-thirds of the total financial expenses incurred in the health sector [16]. Mauritius is no exception. Government hospital services expenditure accounted for 65% of General Government Health Expenditure (GGHE) in the fiscal year 2007/2008 and increased to nearly 70% in 2016 [17, 18]. The rising hospital cost in Mauritius is driven by salaries of health personnel and application of updated diagnostic and therapeutic tools methods to address the high burden of chronic diseases. Rising patients’ expectations and medical errors are inciting health decision makers to implement costly medical diagnostic technologies in major hospitals [19, 20].

GGHE in Mauritius accounts for approximately 2% of GDP and only 10% of General Government Expenditure [21]. This is below the target set under the Abuja 2001 Declaration on health in April 2001, when heads of state of African Union countries pledged to allocate at least 15% of their annual budget to the health sector. In Mauritius advocacy for a higher share of resources allocated to fund hospital services has never subsided. Conversely, as claims for more investment in the health sector with a view to improving better health outcomes abound, the health sector must compete with other public sector priorities to receive its fair share of financial resources. Fiscal space which is the flexibility that the government has within the national budget to augment resources without hampering the financial sustainability and stability of the economy is of essence. There are several means to create fiscal space for the health sector. One of these means is stimulating efficiency with regards to hospitals and health care facilities. The efficiency savings that can be achieved through addressing wastage, and which finds its source in ten main areas of inefficiencies in medicines, human resources and service delivery, can be reinvested into the health sector. Thus, establishing that improving efficiency is inextricably intertwined with fiscal space creation [2, 22].

The objectives of this study are three folds. Firstly, to examine the general trend of hospital efficiency over the period 2001–2017. Secondly, to measure the elasticity of hospital output to changes in inputs variables and finally, to assess the potential scope that improved efficiency can contribute to create fiscal space. There is not much empirical evidence in this area except for a multi-country study in the 45 Sub-Saharan African Countries carried out in 2011. The main findings showed that the mean efficiency score for the 45 countries was 0.70 and the mean potential saving from improved efficiency was 2.02% of GDP per capita in the stochastic frontier analysis (SFA) model. The study revealed that for Mauritius the efficiency score was 0.91 and the potential saving in health expenditure as a result of enhanced efficiency was 0.49% of GDP per capita in the SFA model for the year 2011 [23].

This study is motivated by several considerations. As a high proportion of the recurrent budget of the Ministry of Health and Wellness (MoHW) is spent on hospital services it is vital to maximise returns. Improvements in efficiency level and ultimately productivity of hospitals may result in large savings in healthcare expenditures, which could be devoted to other interventions such as prevention of infectious and emerging diseases. Resource allocation in Mauritius is skewed towards non communicable diseases and based on the current burden of diseases. Infectious and communicable diseases account for approximately 15% of disease burden but the risk of re-emergence of infectious diseases is too important to be ignored. The outbreak of measles in 2018 is a palpable example after several years of interruption. Sustaining financially national programme, including immunisation and HIV are challenges as the funding landscape is becoming uncertain in the context of post COVID-19 pandemic with the domestic economy expected to experience a contraction for the first time in 40 years and forecasted as high as 12.5% [24, 25]. As Mauritius is not eligible for any support under the Global Alliance Vaccination Initiative, investment in the national immunisation programme must be fully funded by MoHW. With regards to HIV an important source of funding is the Global Fund to fight AIDS, TB and Malaria. Since 2008, Mauritius has been a beneficiary of grants from the Global Fund but the funding is been phased down. The forthcoming cycle 2021–2023 of the Global Fund will be the last one for Mauritius to receive financial assistance. Moreover, the global pandemic of COVID-19 inflicting national economies indistinctly calls health financing policymakers to invest adequately in the national programme for emergency preparedness and response with focus on disease surveillance, including large-scale testing and contact tracing. Hospital services are highly labour intensive and a hefty proportion of hospital and specialised health services budget is spent on human resources (approximately 74.8% for fiscal year 2017/2018) [17]. Thus, it is of paramount importance to ensure that human resources are efficiently utilised. In this perspective, it is useful to determine the efficiency of human resources with a view to optimising hospitals contributions to health systems goals, namely improving the health outcomes, responsiveness to patients’ rational expectations, and fairness in financing of care. Finally, efficiency is common to all functions of health systems. Experience of some countries have shown that improving health expenditure efficiency to free fiscal space cannot be played down as the spectre of increasing pressure on government budget looms. Information on efficiency of hospitals will inform decision makers to fulfil the stewardship role of Ministries of Health [4].

## Methods

### Sources of data

The dataset used for this study is unbalanced panel data from the main public regional hospitals over the period 2001–2017 and comprised 41 observations. Disaggregated data at regional hospital level were available from 2001 to 2006. However, as disaggregated data were not available as from 2007, combined data for all the public hospitals were considered. Data were collected from various annual reports of the MoHW and the Ministry of Finance, Economic Planning and Development.

The health care system in Mauritius is a mix of public and private sector providers. Public healthcare delivery is served around a well delineated three tiers system, namely primary, secondary and tertiary. At the apex of the healthcare delivery system are national specialised hospitals and medical centres alongside regional hospitals. The regional hospitals, which are the focus of our study, act as referral centers for a decentralised network of Primary Health Care (PHC) facilities which comprises mediclinics, area and community health centres, within a defined demarcated area and population. The decentralised health care delivery endeavours to ensure minimal dichotomy in terms of access to health services across all regions.

The sampling criteria for selecting hospitals in the study are provision of common wide array of in-patient health care service and an average bed-capacity of at least 80 annually. The sample comprised all five regional public hospitals in Mauritius. The five regional hospitals selected had bed occupancy rate ranging between 62.8 and 80.2% whereas hospital admissions ranged between 82.4 and 86.1% over the period 2001–2017. Moreover, overall the public sector hospitals account for the bulk of bed capacity across Mauritius (85%) and leaving a meagre share to the private sector which comprise a network of 19 health facilities (15%) [26].

Output variables consisted of inpatients admitted and outpatients at the level of Accident, Emergency and unsorted outpatient departments. Inputs variables to the hospital production are measured in terms of capital and labour. Capital inputs are measured in terms of the total number of active hospital beds in all the five main regional hospital. Labour inputs are measured by the sum of personnel employed on a full-term basis in each hospital. To that effect, three categories of staff were considered for the efficiency analysis namely doctors (generalists and specialists), nurses and midwives, and non-medical which included staff other than nurses and doctors. The basis to adopt nomenclature for hospital staff in terms of doctors, nurses and non-medical is based on evidence that each one of them has a distinct role in patient care and deliver medical care at different service levels, especially from a quality and patient satisfaction perspective [27].

### Stochastic frontier analysis (SFA) versus data envelopment analysis (DEA)

The two most commonly used approaches to measure providers’ efficiency are the parametric approach and the nonparametric or deterministic approach. The Stochastic Frontier Analysis **(**SFA) method is mostly used to evaluate parametric stochastic models, unlike the Data Envelopment Analysis (DEA) which is mostly employed to evaluate nonparametric or deterministic models. DEA is applied to analyse hospital efficiency as it is relatively easier to implement given its nonparametric basis, and the freedom provided on the specification of inputs and outputs. The DEA approach circumvents the need to measure output prices which are not available for transactions and services and fee-based outputs [28]. The rationale in favour of SFA developed initially by Aigner et al. [29] and expanded by Battesse and Coelli (1992; 1995) builds around the statistical weaknesses of DEA method [30–32]. The DEA method is non-stochastic. It fails to capture random noise such as dearth of resource inputs resulting from epidemics and strikes. Furthermore, DEA does not allow that statistical tests of the hypothesis regarding magnitude of inefficiencies be performed [33]. The robustness of SFA is based on that it makes a clear distinction between the two sources of error, due to inefficiency and random noise. SFA allows the decomposition of deviations from the efficient frontier into two components, inefficiency and noise [34]. DEA, which cannot distinguish between effects caused by inefficiency and a measurement error, attributes all these effects to inefficiency [35, 36].

SFA can be estimated using the two methods of Maximum Likelihood Estimation and/or Ordinary Least Square in panel data. SFA has the disadvantage that it builds on availability of structured information, including information about the production/cost technology and assumption around the distributional form of the inefficiency term. To that effect the analysis of inefficiencies could well be influenced by the model specification [31]. Albeit, these shortcomings the SFA remains the most reliable approach for measuring hospital inefficiencies. There are two distinct SFA modelling approaches of panel data. The first one generally assume a uniform variation for all production units, as inferred by Battese and Coelli [26, 31]. The others include three stochastic components respectively for efficiency, random noise, and time-invariant heterogeneity [15] and above all assume stochastic variation without any correlation over time [37]. Standard SFA models are limited to only one output and where technical inefficiency is denoted by difference between potential and observed output. Under such situation inpatient and outpatient workload are aggregated into one variable.

### Modelling

Studies carried out to measure hospital efficiency are based mostly on two stochastic production function namely the Cobb-Douglas and the Translog functions. The SFA is a method to estimate a frontier production which assumes a given functional form for the relationship between inputs and an output (Coelli et al. [13]). The general form of the panel data version developed by Aigner, Lovell and Schmidt [29] and the production frontier stated by Coelli, Prasada and Battese (Coelli et al. [38]) is applied to the data as follows:

1 $$ {y}_i=f\ \left(\ {d}_i,{n}_{i,}{m}_i,{b}_i\right)+{e}_i $$

Equation 1 above can be specified as follows:

2 $$ {y}_i={\upbeta}_0+{\upbeta}_1d+{\upbeta}_2n+{\upbeta}_3\ m+{\upbeta}_4b+{e}_i $$

Where y is the output measure (in this case, number of outpatient and inpatient visit), *d* is a vector of doctors employed, n is a vector of nurses employed, *m* is vector of non-medical staff employed, *b* is a vector capital (proxied with number of hospital beds available), *i* is the decision making units (hospitals) and *e* represents errors decomposed as follows in eqs. 3 and 4:

3 $$ {e}_i={v}_i+{u}_i $$

4 $$ y_{i} = {\beta_0} + {\beta_1{d}} + {\beta_2{n}} + {\beta_3{m}} + {\beta_4{b}} + v_{i} + u_{i} $$

Where *v* is a random error term, normally distributed and uncorrelated with the explanatory variables; and *u* represents the hospital specific fixed effects or time invariant technical inefficiency.

For the purpose of this study in addition to the Cobb-Douglas and Translog production functions, a Multi-output distance function will be applied to the dataset.

The Cobb–Douglas form is built on assumptions of constant input elasticities and return to scale for all hospitals. The Cobb Douglas function is represented under eq. 5:

5 $$ \ln \left({y}_{it}\right)={\upbeta}_0+{\sum}_{i=1}^k{\upbeta}_{\mathrm{j}}\ln {x}_{j, it}+\left(\ {v}_{it}-{\mathrm{u}}_{it}\right) $$

Where j is the number of independent variables, i is the hospital), t is the time in years; ln represents the natural logarithm, y_it_ represents the output of the i-th hospital at time t, x_jit_ is the corresponding level of input j of the i-th hospital at time t, β is a vector of unknown parameters to be estimated. The v_it_ is a symmetric random error, accounting for statistical noise with zero mean and unknown variance σv2. The u_it_ is the non-negative random variable associated with technical inefficiency of hospital i, its mean is mi and its variance are σu2 [33].

The Cobb -Douglas function has been transformed to fit the data as illustrated in eq. 6.

6 $$ \ln \left( Outpatien{t}_{it}+ Inpatien{t}_{it}\right)=\beta 0+\beta 1\ln Be{d}_{it}+\beta 2\ln Docto{r}_{it}+\beta 3\ln Nurs{e}_{it}+\beta 4\ln Nonmedica{l}_{it} $$

The translog function relaxes the assumption of constant input elasticities and return to scale for all hospitals but is influenced by degrees of freedom and multicollinearity, as follows:

7 $$\mathrm{In} (y_{it}) = \beta_{o} + \sum^{k}_{j=1}  \beta_{j} \mathrm{In}x_{j,it} +1/2 \sum^{k}_{j=1} \sum^{k}_{j=1} \mathrm{In}x_{h,it} (v_{it} - u_{it})$$

Where j, i, t, ln, y_it,_ x_jit_ are same as under eq. 5 and x_jit_ times x_hit_ is the interaction of the corresponding level of inputs j and h of the i-th hospital at time t, β is a vector of unknown parameters to be estimated. The *v*_*it*_ is a symmetric random error, to account for statistical noise with zero mean and unknown variance σv2. The *u*_*it*_ is the non-negative random variable associated with technical inefficiency of hospital i, its mean is mi and its variance is σu2 [33].

The Translog function form as defined in eq. 7 above is transformed as follows to suit the purpose of the current study.

8 $$ {\displaystyle \begin{array}{l}\ln \left({\mathrm{Outpatient}}_{\mathrm{it}}+{\mathrm{Inpatient}}_{\mathrm{it}}\right)=\upbeta 0+\upbeta 1\ {\mathrm{lnBed}}_{\mathrm{it}}+\upbeta 2\ {\mathrm{lnDoctor}}_{\mathrm{it}}+\upbeta 3\ {\mathrm{lnNurse}}_{\mathrm{it}}+\upbeta 4\ {\mathrm{lnNonmedical}}_{\mathrm{it}}\\ {}\kern12em +\upbeta 12\ \left({\mathrm{lnBed}}_{\mathrm{it}}\times {\mathrm{lnDoctor}}_{\mathrm{it}}\right)+\upbeta 13\ \left({\mathrm{lnBed}}_{\mathrm{it}}\times {\mathrm{lnNurse}}_{\mathrm{it}}\right)\\ {}\kern12em +\upbeta 14\ \left({\mathrm{lnBed}}_{\mathrm{it}}\times {\mathrm{lnNonmedical}}_{\mathrm{it}}\right)+\upbeta 23\ \left({\mathrm{lnDoctor}}_{\mathrm{it}}\times {\mathrm{lnNurse}}_{\mathrm{it}}\right)\\ {}\kern12em +\upbeta 24\ \left({\mathrm{lnDoctor}}_{\mathrm{it}}\times {\mathrm{lnNonmedical}}_{\mathrm{it}}\right)\\ {}\kern12em +\upbeta 34\left({\mathrm{lnNurse}}_{\mathrm{it}}\times {\mathrm{lmNonmedical}}_{\mathrm{it}}\right)\\ {}\kern12em +\upbeta 11\ 0.5\ \left({\mathrm{lnBed}}_{\mathrm{it}}\times {\mathrm{lnBed}}_{\mathrm{it}}\right)+\upbeta 22\ 0.5\left({\mathrm{lnDoctor}}_{\mathrm{it}}\times {\mathrm{lnDoctor}}_{\mathrm{it}}\right)\\ {}\kern12em +\upbeta 33\ 0.5\ \left({\mathrm{lnNurse}}_{\mathrm{it}}\times {\mathrm{lnNurse}}_{\mathrm{it}}\right)\\ {}\kern12em +\upbeta 44\ 0.5\ \left({\mathrm{lnNonmedical}}_{\mathrm{it}}\times {\mathrm{lnNonmedical}}_{\mathrm{it}}\right)\end{array}} $$

Where, β0 is the intercept of the constant term, β1, β2, β3, β4 are first order derivatives, β11, β22, β33, β44 are own second order derivatives and β12, β13, β14, β23, β24, β34, are cross second order derivatives. In view that the double log form model (with both the dependent and explanatory variables been in natural logs), the estimated coefficients show elasticities between dependent and explanatory variables [30, 33].

Finally, the rationale for using a Multi-output distance function is that the specified model of hospital production and inefficiency can be ran without aggregating inpatient and outpatient visits. The Multi-output distance function adapted to the current research is shown in eq. 9 below.

9 $$ {\displaystyle \begin{array}{l}\ln \left({\mathrm{Outpatient}}_{it}\right)=\upbeta 0+\upbeta 1\ {\mathrm{lnBed}}_{\mathrm{it}}+\upbeta 2\ {\mathrm{lnDoctor}}_{\mathrm{it}}+\upbeta 3\ {\mathrm{lnNurse}}_{\mathrm{it}}+\upbeta 4\ {\mathrm{lnNonmedical}}_{\mathrm{it}}\;\\ {}\kern7em +\upbeta 12\ \left({\mathrm{lnBed}}_{\mathrm{it}}\times {\mathrm{lnDoctor}}_{\mathrm{it}}\right)+\upbeta 13\ \left({\mathrm{lnBed}}_{\mathrm{it}}\times {\mathrm{lnNurse}}_{\mathrm{it}}\right)\\ {}\kern7em +\upbeta 14\ \left({\mathrm{lnBed}}_{\mathrm{it}}\times {\mathrm{lnNonmedical}}_{\mathrm{it}}\right)+\upbeta 23\ \left({\mathrm{lnDoctor}}_{\mathrm{it}}\times {\mathrm{lnNurse}}_{\mathrm{it}}\right)\\ {}\kern7em +\upbeta 24\ \left({\mathrm{lnDoctor}}_{\mathrm{it}}\times {\mathrm{lnNonmedical}}_{\mathrm{it}}\right)\\ {}\kern7em +\upbeta 34\left({\mathrm{lnNurse}}_{\mathrm{it}}\times {\mathrm{lmNonmedical}}_{\mathrm{it}}\right)\\ {}\kern7em +\upbeta 11\ 0.5\ \left({\mathrm{lnBed}}_{\mathrm{it}}\times {\mathrm{lnBed}}_{\mathrm{it}}\right)+\upbeta 22\ 0.5\left({\mathrm{lnDoctor}}_{\mathrm{it}}\times {\mathrm{lnDoctor}}_{\mathrm{it}}\right)\\ {}\kern7em +\upbeta 33\ 0.5\ \left({\mathrm{lnNurse}}_{\mathrm{it}}\times {\mathrm{lnNurse}}_{\mathrm{it}}\right)\\ {}\kern7em +\upbeta 44\ 0.5\ \left({\mathrm{lnNonmedical}}_{\mathrm{it}}\times {\mathrm{lnNonmedical}}_{\mathrm{it}}\right)\\ {}\kern7em +\upbeta 5\ {{\mathrm{lnY}}^{\ast}}_{\mathrm{it}}+\upbeta 51\ \left({\mathrm{lnBed}}_{\mathrm{it}}\times {{\mathrm{lnY}}^{\ast}}_{\mathrm{it}}\right)+\upbeta 52\ \left({\mathrm{lnDoctor}}_{\mathrm{it}}\times {{\mathrm{lnY}}^{\ast}}_{\mathrm{it}}\right)\\ {}\kern7em +\upbeta 53\ \left({\mathrm{lnNurse}}_{\mathrm{it}}\times {{\mathrm{lnY}}^{\ast}}_{\mathrm{it}}\right)+\upbeta 54\ \left({\mathrm{lnNonmedical}}_{\mathrm{it}}\times {{\mathrm{lnY}}^{\ast}}_{\mathrm{it}}\right)\\ {}\kern7em +\upbeta 55\ 0.5\ \left({{\mathrm{lnY}}^{\ast}}_{\mathrm{it}}\times \mathrm{lnY}{\ast}_{\mathrm{it}}\right)\end{array}} $$

where, Y* is the ratio of outpatient visits to inpatient admissions. β5, is the first order derivative while β55 represents own second order derivative. β51, β52, β53, β54, are cross second order derivatives [33].

The Cobb-Douglas, Translog and Multi-output distance function models were estimated using STATA 11.

### Technical efficiency assessment

Adapting the common widely accepted definition for technical efficiency to the present study the ratio of the observed output (Y_*it*_) to the maximum feasible output (Y_max_), defined by a certain level of inputs used by the hospital will determine the level of technical efficiency of hospital *i* at time *t.* Since both the Cobb Douglas and Translog functions are based on a normal-truncated normal maximum likelihood (ML) random model effect with time invariant efficiency developed by Battese and Coelli [38], the maximum feasible output is determined by the hospitals with inefficiency effect equal to 0 (*v*_*it*_ = 0). Technical efficiency is derived building on the premises stated earlier under eqs. (1) and (3) where the general form of the panel data version is.

10 $$ \ln {y}_{it}=f\left({x}_{j, it},\beta \right)+{e}_i $$

Equation (10) can be formulated as:

11 $$ {y}_{it}=\exp \left(f\left({x}_{j, it},\kern0.5em \beta \right)\right)\ast \exp \left({v}_{it}\right)+\exp \left(-{u}_i\right) $$

Where f() is a suitable functional form of any of the model namely Cobb-Douglas, Translog and Multi-output distance, y_it_ represents the output of the i-th hospital at time t, x_j,it_ is the corresponding level of input j of the i-th DMU (hospital) at time t, and β is a vector of unknown parameters to be estimated [33].

The technical efficiency can be expressed as follows:

12 $$ T{E}_{it}=\frac{y_{it}}{e\mathrm{xp}\left(f\left({x}_{j, it},\kern0.75em \beta \right)\right)\ast \exp \left({v}_{it}\right)} $$

*or*

13 $$ T{E}_{it}=\frac{e\mathrm{xp}\left(f\left(\ {x}_{j, it},\kern0.75em \beta \right)\right)\ast \exp \left({v}_{it}\right)+\exp \left(-{u}_i\ \right)\ }{e\mathrm{xp}\left(f\left({x}_{j, it},\kern0.5em \beta \right)\right)\ast \exp \left({v}_{it}\right)} $$

14 $$ T{E}_{it}=E\ \left[e\left(-{u}_{it}\right)/\left({v}_{it}-{u}_{it}\right)\right] $$

Where *u*_*it*_ represents hospital specific fixed effects or time invariant technical inefficiency and *v*_*it*_ is a normally distributed random error term and is uncorrelated with the explanatory (independent) variables. Technical inefficiencies range between 0 and 1 as *u*_*it*_ is a nonnegative random variable. A value of 0 infers that hospital is technically inefficient and, conversely, a value of unity implies perfect technical efficiency. Technical efficiency is calculated following Battese and Coelli, 1995, using the bc option available in STATA 11.

### Quantification of potential gains through technical efficiency

As all public health facilities provides free health care services the sole source of revenue is annual government budgetary allocation. Potential gains from technical efficiency for all the five regional hospitals in this study are estimated by determining the share of annual government budgetary allocation received that could be saved potentially should technical inefficiency be avoided. The share of grants revenue that can be saved is defined in eq. (15).

15 $$ \mathit{\operatorname{Re}}{v}_i=\left( ef{f}_{max}-{eff}_i\right)\ast {G}_i $$

Where *Rev*_*i*_ represents annual budgetary allocation of the i-th hospital that could be saved if inefficiencies were eliminated, *eff*_*max*_ is maximum efficiency level (1.00 in this case), *eff*_*i*_ is the current efficiency score of the i-th hospital estimated under the SFA specification above (translog function) and *G*_*I*_ is the actual budget allocated by the government to the i-th hospital [41].

The savings realised in total hospital budgetary grant allocation (*Rev*_*i*_) is a proxy of the potential fiscal space available for the i-th hospital in case full efficiency is attained [22].

## Results

### Descriptive statistics

Table 1 provides a descriptive statistic of the variables, both inputs and outputs, used in the study over the period 2001–2017. The annual inpatient admissions and outpatient visits for all hospitals selected were about 187,396 and 1,453,652, respectively. An annual average number of 984 doctors, 3184 nurses, and 3216 non-medical staff were noted.

**Table 1:** Descriptive statistics of the input and output variables

Variable	Average value, annually	Standard deviation	Minimum	Maximum
***Number of beds***	3216	438	2260	3699
***Number of doctors***	984.	170	808	1514
***Number of nurses***	3184	278	3051	4016
***Number of non medical staff***	3216	438.1	2560	3699
***Number of outpatients***	1,453,652	98,042	1,198,329	1,604,317
***Number of inpatients***	187,396	20,767	151,520	212,520
***Average length of stay***	3.08	.32	2.6	3.58
***Number of hospital days***	638,256	60,326	563,386	735,385
***Bed occupancy rate***	0.8155	0.0480	0.7565	0.9214

Source (MOHW 2001–2017)

**Fig. 1:**
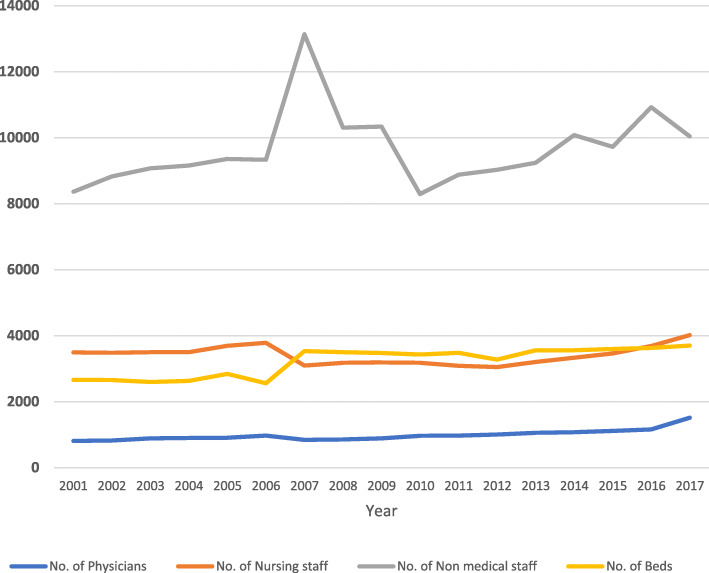
Trends of the number of doctors, nurses, non-medical staff and beds, 2001 to 2017, in public hospitals

**Fig. 2:**
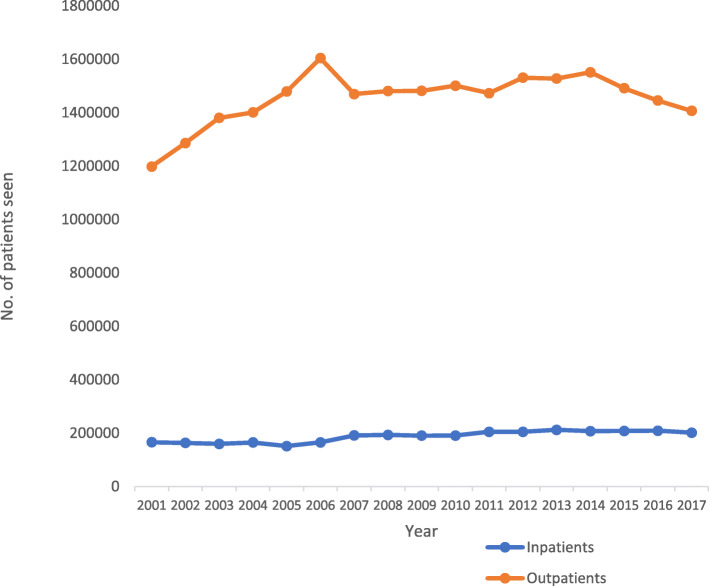
Trends of the number of inpatients and outpatients, 2001 to 2017, in five regional public hospitals

Figures 1 and 2 illustrate the number of resources over the study period. Figure 1 shows a general rising trend for the number of doctors and beds over the period 2001 to 2017. In contrast, there were some fluctuations in the number of nurses and nonmedical staff but overall the trend was upward (Fig. 1). The number of inpatients visits and outpatient admissions for the hospitals increased by 22 and 17%, respectively (Fig. 2).

### Stochastic production frontier estimates

Estimates of the SFA functions are presented in Table 2. The upper section of the table presents results for the distinct parameters of the production functions (four parameters for Cobb–Douglas function, 14 parameters for Translog function and 20 parameters for Multi-output function). The lower part of the table shows the variance parameters, the amount of the function of the log likelihood, and the Likelihood Ratio test.

**Table 2:** Maximum likelihood estimates of the stochastic frontier models (*n* = 41)

Ln (output)	Parameter	Cobb–Douglas function	Translog function	Multi-Output distance
Constant	β0	5.274****	2.904****	6.539**
Ln (bed)	β1	0.232****	*0.171*	*0.411*
Ln (doctor)	β2	0.618****	11.756****	13.629****
Ln (nurse)	β3	0.165	−8.156****	−9.422****
Ln (non-medical staff)	β4	0.228 ^***^	*0.249*	−0.311*
Ln (bed) × ln (doctor)	β12		*0.444*	0.385*
Ln (bed) × ln (nurse)	β13		*0.600*	*1.052*
Ln (bed) × ln (non-medical staff)	β14		*−0.433*	*− 0.409*
Ln (doctor) × ln (nurse)	β23		−4.62****	1.107**
Ln (doctor) × ln (non-medical staff**)**	β24		*0.966*	1.485****
Ln (nurse) × ln (non-medical staff**)**	β34		*−0.2*	*0.777*
Ln (bed) × ln (bed)	β11		*−0.461*	0.989**
Ln (doctor) × ln (doctor)	β22		1.88****	3.3785*
Ln (nurse) × ln (nurse)	β33		4.343*	*4.24*
Ln (non-medical staff**)** × ln (non-medical staff)	β44		*−0.355*	*−1.005*
Ln (outpatient/inpatient)	β5			−2.85****
Ln (outpatient/inpatient) × ln (bed)	β51			*0.064*
Ln (outpatient/inpatient) × ln (doctor)	β52			1.084****
Ln (outpatient/inpatient) × ln (nurse)	β53			0.677*
ln (outpatient/inpatient) × ln (non-medical staff)	β54			*−0.270*
Ln (outpatient/inpatient) x ln (outpatient /inpatient)				*0.010*
Variance of technical inefficiency (sigma_u2)	δu2	*0.018*	*6.41 e − 06*	*5.04e-07*
Variance of random error (sigma_v2)	δv2	*0.004*	*0.0017*	*.0013*
Sigma square (sigma2)	δs2 = δu2 + δv2	*0.022*	*0.0017*	*.0013*
Ln sigma square (lnsigma2)	Ln (δs2)	−3.780****	*−6.35*	*−6.57*
Variance ratio parameter (gamma)	ϒ = δu2/δs2	*0.79*	*0.0036*	*0.00015*
Inverse logit gamma (ilgtgamma) = 0	ilgt ϒ	*1.325*	*−5.600*	*−7.92*
mu	μ	*0.045*	*−0.0949*	*−.0266*
Wald Chi square (3)	χ2	*887.41*	*489*	*135*
Number of observations	N	*41*	*41*	*41*
Log likelihood		*44*	*70*	*75*

* *p* < 0.08; ** *p* < 0.05; *** *p* < 0.01; **** p < 0.001

The Maximum LIkelihood estimates of stochastic frontier production function are based on a normal-truncated normal random-effects model with time-invariant efficiency developed by Battese and Coelli [38]. Comparing which of the two most widely applied functional forms for the stochastic frontier model (Translog or the Cobb-Douglas) fits the data better, the null hypotheses that the multiplicative terms in the Translog function are simultaneously equal to zero (H0 = β11 = β22 = β33 = β12 = β13 = β14 = β23 = β24 = β34 = 0**)** is tested. Using the likelihood ratio test, the Translog functional form (Likelihood Ratio = 70.7, *P* < 0.001 was found to be more appropriate compared to that of Cobb-Douglas functional form [32, 33].

The nature of variations in inefficiency whether are random or systematic lies in the value of ϒ under Table 2. On a scale ranging from zero to one, the lower the value of gamma ϒ, the greater differences in the production will be associated to statistical noise. In the same breadth, the closer gamma ϒ is to one implies presence of technical inefficiency. The low estimated value of parameter ϒ (0.0036), suggests only a meagre 0.36% of the total variation of total production can be attributed to inefficient error term and 99.64% of the total variation linked to the stochastic random errors. In a nutshell, the variation of the total production among the different hospitals cannot be explained by differences in their respective production inefficiencies.

The fact that interactions of beds with doctors, nurses and non-medical staff were not statistically significant, raise some reservations on the presence of outpatients as part of output as it may well subside the importance of hospital beds in terms of hospital outputs.

### Output elasticities of resource inputs

Though the Translog model is a better fit and representation of the data than Cobb-Douglas, a challenge is that the first order coefficients are not that decisive as not much can be inferred of the impact of the input variables (beds, doctors and nurses) on output (inpatients admitted and outpatients seen). Consequently, to determine the real impact of each input distinctly, that is how hospital outputs respond to changes in the input variables, the marginal effects were calculated using Eq. (16) wherein the marginal product is equal to the elasticity of scale for each input.

16 $$ \kern1.5em e\ \mathrm{i}\kern0.5em =\kern0.75em \updelta\ \ln \frac{\left(y\mathrm{i}\right)}{\left(x\mathrm{ji}\ \right)}=\kern0.5em {\sum}_{j=1}^4{\upbeta}_{\mathrm{jh}}\ln {x}_j\kern0.5em +\kern1em {\upbeta}_{\mathrm{jt}} $$

The respective output elasticities of each of the inputs, based on equation above, are shown in Table 3.

**Table 3:** Output elasticities of input variables (Scale elasticity)

Inputs	Scale elasticity
Number of beds	0.51
Number of doctors	- 0.24
Number of nurses	0.73
Number of nonmedical staff	0.16
**Total**	**1.16**

The output elasticities which measure the responsiveness of output to a unit change in inputs show that a unit increase in the number of nurses, beds and non-medical staff will lead to an increase in outputs by 0.73 unit,0.51 unit and 0.16 input, respectively. Conversely, a unit increase in the number of doctors will lead to a fall in output by 0.24 unit. The aggregates of these elasticity coefficients sum to 1.16, that is if all hospitals increase their inputs by 1% outputs will increase by 1.16%. Thus, inferring that the output process, in terms of patients treated, has increasing returns to scale and that the five regional public hospitals have been operating at the optimum production scales. Furthermore, increasing the inputs by 1% with exception of doctors which is held constant, will result in an increase in hospital output by 1.4%.

### Technical efficiency

Technical efficiency was estimated using a one-step maximum likelihood estimates procedure for all three functions (Cobb -Douglas, Translog and Multi-output distance). As disaggregated data for the inputs at hospital level were only available over the period 2001 to 2006, Fig. 3 illustrates the trend over the said period for both Translog and Multi-output distance functions. During the period 2001 to 2006, all five regional hospitals achieved full technical efficiency at least once under the Translog function. Likewise, under the Multi-output function, except for AG Jeetoo Hospital, the other hospitals achieved full technical efficiency at least once. Table 4 illustrates the score of technical efficiencies for all regional hospitals combined for the three functional forms covering the entire period 2001–2017. Under the Translog model, the mean efficiency of the hospitals was 0.841, which indicated an average wastage of resources as high as 15.9%. Results from the Multi-distance output function inferred that the mean technical efficiency was slightly higher amounting to 89%. Both functional models imply there is room for more efficiency as regional hospitals employ lesser resources to produce the current output levels.

**Fig. 3:**
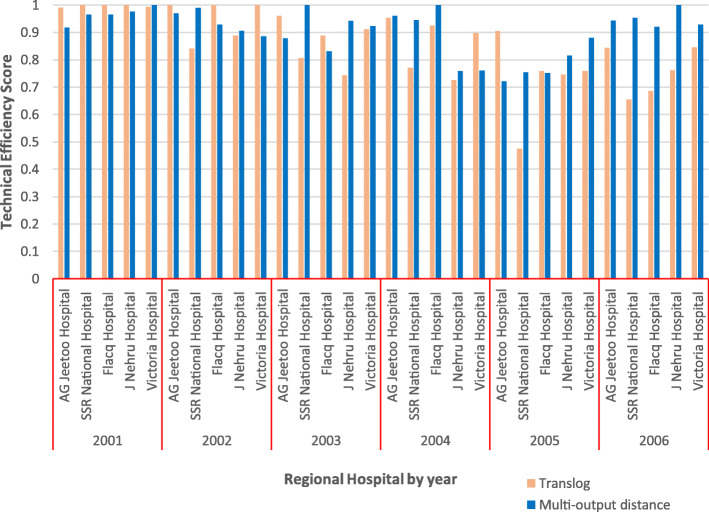
Technical Efficiency by major Regional Hospital using Translog and Multi-output distance functions, 2001–2006

**Table 4:** Technical efficiency scores

Function	Technical efficiency	Standard deviation	95% Confidence interval
Cobb Douglas function	0.83	0.13	0.64 0.97
Translog function	0.84	0.15	0.79 0.89
Multi-output function	0.89	0.10	0.86 0.92

In order to test that the mean difference of the technical efficiency score between the Translog and Multi-output distance functions was statistically significant a paired t-test was run for the period 2001–2017. Technical efficiency of hospitals was slightly lower under the Translog function (0.84 + 0.16) compared to the Multi output distance function (0.89 + 0.10). A statistical increase of 0.05 (95% Confidence Interval: 0.008–0.09) with t-value been 2.4 was obtained, indicating that the t-statistics is significant. Thus, inferring that there is much support for rejecting the hypothesis that there is no difference in the mean of the Translog function and Multi-output distance function or accepting the alternative hypothesis that the mean of the two functions differ.

It is worth underlying that the mean annual technical efficiency for all the regional hospitals using the Multi-output distance function has been on the decline over the period 2012 to 2016, from 0.89 to 0.71. The mean technical efficiency for the period 2012–2017 (0.81) is lower compared to that of the period 2001–2017(0.89).

### Potential savings from achieving technical efficiency

Table 5 provides the potential savings that can be achieved based on improvements from the mean efficiency level (estimated at 0.89 for the period 2000–2017 and 0.81 for the period 2012–2017) under three different efficiency score scenarios over period 2019 to 2021. The three scenarios considered are achieving efficiency scores of 0.9, 0.95 and 1.0.

**Table 5:** Forecasted average potential savings arising from improved efficiency, 2020–2022, under three scenarios

	Fiscal year 2020–2021 ^**a**^		Fiscal year 2021–2022 ^**b**^				Fiscal year 2022–2023 ^**c**^		
	Efficiency score (0.90)	Efficiency score (0.95)	Efficiency score (1 .0)	Efficiency score (0.90)	Efficiency score (0.95)	Efficiency score (1.0)	Efficiency score (0.90)	Efficiency score (0.95)	Efficiency score (1.0)
Potential savings based on mean efficiency of 0.89 (2000–2017)									
MUR million	99	592	1085	106	633	1161	107	643	1180
US$ million	2.5	15.1	27.7	2.7	16.2	29.6	2.7	16.4	30.1
As a % of GGHE	0.8	5.1	9.3	0.8	4.8	8.9	0.8	5.0	9.2
Potential savings based on mean efficiency of 0.81 (2012–2017)									
MUR million	888	1381	1874	950	1478	2005	965	1501	2038
US$ million	22.6	35.2	47.8	24.2	37.7	51.2	24.6	38.3	52.0
As a % of GGHE	7.6	11.8	16.0	7.3	11.3	15.3	7.5	11.7	15.9

^a^ Hospital Services Expenditure Fiscal year 2020 -2021 (MUR 9862 million)

^b^ Hospital Services Expenditure Fiscal year 2021–2022 (MUR 10,555 million)

^c^ Hospital Services Expenditure Fiscal year 2021–2022 (MUR 10,724 million)

The estimates show that, assuming a mean efficiency of 0.89, hospital services could save between MUR 663 million (US$ 16.2 million) to MUR 1161 million (US$ 29.6 million) in fiscal year 2021–2022 should inefficiencies be addressed such that the efficiency score is improved to 0.95 and 1.0, respectively. These represent significant additional fiscal space from within the available resource envelope. More specifically in fiscal year 2021–2022, an increase in efficiency score to 0.95 would account for an additional fiscal space (expressed as a share of GGHE) of 4.8% and as high as 8.9% in case full efficiency is achieved.

Assuming that in 2020–2021 the mean efficiency is 0.81, as estimated for the period 2012–2017, hospital services could save between MUR 1478 million (US$ 37.7 million) to MUR 2005 million (US$ 51.2 million) in fiscal year 2021–2022 should inefficiencies be tackled such that the efficiency score is improved to 0.95 and 1.0, respectively.

## Discussion

An important caveat is that findings of the study are based on the choice of inputs and outputs. The policy implications that these entail here should be considered within this perspective.

The first order coefficients for both number of beds and non-medicals are positive but the coefficient of interaction between these two input variables is negative. The number of beds has two effects, direct and indirect, on hospital outputs. The number of beds directly and positively affects output. Furthermore, the number of beds indirectly influences the effect of number of doctors, nurses, and non-medical staff on the output. As interaction between beds and non-medicals is negative, this prompts some degree of substitution between non-medical and hospital beds. The Translog and Multi-output distance functions conclude that 1% increase in number of beds would reduce the number of non-medical required ranging between 0.41 and 0.43%. This means that should there be more beds than required, productivity of non-medicals could be reduced leading to lower output level.

The second order coefficients and interaction terms coefficients under the Translog and Multi-output distance functional forms are statistically significant for doctors, nurses and interaction between doctors and nurses. The high significance of the interaction between doctors and nurses suggests that these two inputs are interdependent on each other. The interaction between beds and doctors was, also, found to be significant under the Multi-output distance function. In the same vein, under the Translog functional form doubling or squaring the use of doctors and nurses which in other words means using these inputs once again, implies that the number of doctors and nurses would increase hospital output by 1.88 and 4.34 units per unit of output, respectively. A similar finding is noted under the Multi-output distance functional form. This confirms that investment in doctors and nurses promotes increasing return to scale for both functional forms. The coefficient of number of doctors been positive and significant, as well as for the coefficient of the square of number of doctors, implies that increasing doctors in hospital will contribute to improving the output. With regards to nurses, while the coefficient is negative but significant and the coefficient of the square of number of nurses is significant and positive, infers that doubling the number of nurses would lead to important improvement of hospital output under both functional forms. While health care delivery at the hospital involves many other tasks than just the direct interaction of doctors and patients, the findings highlight the importance of nurses. A 1% increase in number of nurses would reduce the number of doctors and non-medical required by 4.62 and 0.2%, respectively.

Under the Multi-output distance function the first order coefficients for doctors and nurses are positive and negative, respectively. An increase of 1% in number of doctors would result in an increase in hospital output of 13.6% but this is mitigated by 9.4% should there be a rise of 1% in the number of nurses employed. Doctors and nurses are observed to be interdependent and complement each other as demonstrated by the positive sign observed for the interaction between both input variables. The number of doctors has two pronged effects on hospital output. Through the direct effect, the number of doctors impact positively on hospital output, whereas indirectly number of doctors influences the effect of number of nurses on output. Results also show that a 1% increase in number of doctors would increase the number of nurses required by 1.1%.

A general rising trendline for non-medical staff employed in the five regional hospitals over the period 2001–2017 has been noted while the technical efficiency under both functional forms has been declining. The results clearly show that while the coefficient of number of non-medical staff is significant and negative the coefficient of the square of number of nonmedical staff is significant and positive. This infers that hospitals with lower number of non-medical staff are more productive that hospitals with higher number of non-medical staff. A decrease in the number of nonmedical staff in each hospital will result in the improvement of production. It is, therefore, essential that mix of non-medical staff for each regional hospitals within the general workforce be regularly assessed and reviewed accordingly.

The mean technical efficiency estimates of public regional hospitals in Mauritius, under the Multi-output distance function, compares favourably with those in Netherlands and Saudi Arabia and which are 0.84 and 0.846, respectively [39, 40]. In the same vein, compared to hospitals in 45 sub Saharan Africa countries, the public regional hospitals in Mauritius are generally more efficient [27]. A word of caution when interpreting efficiency is that a score of 0.89 does not mean that the regional hospitals can be made more efficient by 11%. It implies that compared to the estimated frontier, efficiency can be improved by 11%. Also, savings made from improved efficiency should be viewed as an alternative for additional resource investment in the health sector. The financial savings complement partners’ endeavours to boost investment for better health service delivery [41].

Evidence abounds that efficiency improvements and resource savings, constitute a critical source of fiscal space for the health sector [22, 42]. The findings confirm that notwithstanding regional public hospitals have relatively high technical efficiency scores, the potential for additional fiscal space to be created through improved efficiency is substantial and cannot be overlooked. This current study estimates that potential saving achieved through improved efficiency represents approximately 0.3% of GDP per capita in 2018, compared to 0.49% in a study in Sub Saharan African countries using 2011 data [20]. The latter study, also, estimated efficiency at 0.91 in 2011 while under this study the mean efficiency score in 2011 at 0.89. Thus, only 0.02 percentage point difference between the two studies.

The financial savings that are estimated to be achieved should all hospital inefficiencies be eliminated (ranging between US$ 29.6 million and US$ 51.2 million in fiscal year 2021–2022) are more than enough to fund entirely the national HIV response, sustaining the expanded programme for immunization and the strengthening emergency preparedness and response programme. This financial contribution of improved efficiency may seemed rather trivial but against the backcloth of phasing out of any financial assistance from the Global Fund for AIDS, Tuberculosis and Malaria by 2023, the non-eligibility of Mauritius to access the Global Alliance Vaccine and Immunization and increasing pressure to build core competencies to meet International Health Regulations [43], daunting challenges are lurking ahead. The annual estimated cost of funding the national response for HIV is US$ 7 million focusing on the key affected population in view that the epidemic is a concentrated one. The cost of funding immunization within the public health services is expected to grow from US$ 2.7 million as per expenditure incurred in 2017 to US$ 5.7 million in 2021. The immunization cost is driven by vaccines at 65% followed by service delivery which includes all activities designed to get the vaccine to the clients such as salaries, transportation, cold chain maintenance and overhead [44]. Furthermore, as COVID-19 pandemic is hitting Mauritius with a double shock in terms of public health and the economy, any fiscal space that can be created in the short to medium term will be an important breath of fresh air for financing the health sector. Until a vaccine or effective treatment become viable solutions to halt the transmission of COVID-19 and associated hospitalisation, investments in disease surveillance, including large-scale testing and contact tracing, are unforeseen emerging priorities. The increasing reliance on funding from government revenue can be eased through fiscal space generated.

The mean technical efficiency over the period 2012–2017 is estimated at 0.81 and lower compared to the mean of 0.89 for the period 2001–2017. Applying a mean technical efficiency score of 0.81 to the estimated public health expenditure for fiscal year 2020–201 and assuming full technical efficiency is achieved in fiscal year 2022–2023, the fiscal space created would amount to MUR 2038 million (US$ 52 million). Table 5, also, provides the potential savings that can be achieved assuming that baseline technical efficiency level of 0.81 increase incrementally to a situation of full efficiency over the period 2020–2023. The financial savings, should full efficiency be achieved, also represents 15.3 to 15.9% of GGHE over the period 2020–2023. The financial savings are significant as current projections estimate that the aftermath of COVID-19 pandemic on the domestic economy will be a contraction of 12.5% in 2020 [24, 25]. The economic contraction will impact negatively on the tax base and consequently reduce substantially the existing leverage to sustain current recurrent expenditure and capital investment in the public health system beyond fiscal year 2020–2021.

A common feature of past and ongoing health sector reforms in Mauritius has been to improve efficiency in service delivery. To that effect, pursuit of decentralization of health services and strengthening PHC have been the focus. However, these seemed not to have been translated into improved efficiency and productivity. Attendances at PHC facilities across Mauritius have risen on average by 4% over the period 2013–2018 but nevertheless these first point of care remain relatively under-utilised. It is estimated that in 2017 and 2018 on average 54% of cases seen at hospital level could have been cared at the PHC facilities. The findings also throw more weight to proponents’ arguments to enhance PHC as the gateway to the health sector. Minimising duplication of services at PHC and hospitals, coupled with a reinforcement of the role of regional hospitals in promoting coordination between PHC and tertiary care institutions would go a long way to enhance hospitals efficiency performance. There is currently no formalised system requiring patients to be registered with a PHC facility. The fact patients are free to attend several different hospitals and receive multiple prescriptions for the same problem needs to be addressed. Patients bypassing the PHC is a palpable source of hospital inefficiency and not to mention adverse medication interactions these might entail. In 2018 as much as 62.5% of patients’ attendances were seen at hospital level while only 37.5% at PHC facilities [26]. The motivations for bypassing needs to be thoroughly studied and addressed promptly. Ensuring continuum of care through a life course approach as well as rolling out the e-health project envisaged since a few years to ensure adequate tracking of the patients’ journey across the health system are urgent panacea. As the elasticity of doctors with respect to outputs, in terms of patients treated is − 0.24, deducing that is a 1% increase in number of doctors would reduce hospital outputs by 0.24%, calls for shifting of doctors from hospitals to health centres to strengthen delivery of PHC at peripheral first point of contact and care.

According to WHO report (2016) use of core hospital resources, namely, doctors, nurses, and beds, below suboptimal level reduces the demand for health services and hampers efficiency [45]. In the same vein, other studies done in high income countries revealed that operating at an optimum sizes and bed numbers at hospital levels can maximise responsiveness of hospitals to population needs and, ultimately, increase efficiency. Evidence generally shows that the optimum efficiency level of a hospital in terms of hospitals beds is when active beds varies between 200 and 300. Furthermore, hospitals with less than 200 beds or more than 600 beds have higher costs [46–49]. Based on 2018 statistics, two of the five regional hospitals had more than 600 beds on average daily and suggesting likelihood of high costs and inefficiency.

While international standards state that the threshold level for optimal efficient of hospital resources is a bed occupancy rate between 84 and 85%, there is room for improvement on the basis that the average occupancy rate for the regional hospitals over the period 2001–2017 was 81.5% [50]. A more focus analysis reveals that bed occupancy rate for the period 2013–2018 has increased for three regional hospitals while for the other two remaining ones a significant drop was noted. Thus, an average drop in beds occupancy rate for all five hospitals combined was estimated from 76.7% in 2013 to 75.6% in 2018. This excess capacity at two of the five regional hospitals suggest, policy makers to consider two possible options. First, convert the excess beds and space to provide day care outpatients secondary prevention services. Second, envisage renting excess beds and space to private medical practitioners, in case there is a demand. However, overall across all the public sector hospitals, the findings show that excess capacity on average for the period 2001–2017 has not yet been reached as a 1% increase in number of beds can still result in a 0.51% rise in outputs.

The coefficient of the interaction between doctors and hospital beds under both the Translog and Multi-output distance functions, being positive and statistically significant, implies both resource input variables are complementary. An increase in the number of beds would increase the number of doctors required by 0.38%. This implies that with increases in hospital beds the productivity of doctors will rise. Thus, doctors may be inclined to keep patients hospitalised of lesser duration, thereby freeing beds, which will increase the hospital outputs in terms of higher number of patients treated.

Whilst improving hospital efficiency is acknowledged as a source of fiscal space creation on the other hand levying exercise taxes on tobacco and alcohol is another viable option. In fact, taxation represents a progressive source of financing the health sector which implies no efficiency cost and furthermore not influenced by the health or income of its population [51]. It is estimated that revenue raised through excise taxes on tobacco and alcohol products accounted for nearly 84% of GGHE in the fiscal year 2018–2019. As regards tobacco products and given its high price inelasticity, increases in the rate of excise taxes will affect consumption proportionally less that increase in taxes. Consequently, government revenue will continue to increase. Valdois et al. [52] examined three scenarios with increases in tobacco excise taxes by 30% in fiscal year 2019–2020 followed by increases of 20% in successive fiscal years 2020–2021, 2021–2022 and 2022–2023. The projections reveal an increase of 69% in government revenue over the period 2019 and 2023. As there has been no increase in exercise taxes on cigarettes since fiscal year 2018–2019 the scenario to increase additional revenue through excise taxes is still valid. Implementing an increase of 30% in excise taxes, including Value added taxes, on cigarettes will create additional revenue of 18.58%. This fiscal space creation represents on average 8% of GGHE [52].

Dearth of capacity to make adequate use of information for policy analysis and formulation of strategic planning to legislate, regulate and enforce good practices hampers achievement of efficiency. In the same vein, as medicines and human resources for health are the two key cost drivers in Mauritius, the lack of an adequate national policy guiding both is a barrier for the three tiers of the public health system to be fully efficient. It is imperative that policy makers in Mauritius develop and implement evidence-based national policies for medicines and human resources for health for the purpose of improving efficiency.

With NCDs accounting for over 85% of the diseases burden and a rapidly ageing population the challenge facing the national health system is to cope with ramping patients’ expectations for quality health care and scale up efficiency across all levels of health care delivery, including hospitals.

The need for creating fiscal space is vital as Mauritius embraces the principles of a welfare state with a national policy of free health care for all in all government-owned health facilities. In 2014, as high as 72.8% of health care services (inpatient, outpatient and day care) were accessed through the widespread network of public health facilities [53]. Paradoxically, Out-of-pocket health expenditure is high accounting for 48.9% of Current Health Expenditure in 2017 [21]. The proportion of population facing catastrophic health expenditure due to out-of-pocket payments rose from 5.78% in 2001/02 to 8.85% in 2012 [54]. The phenomena of rising out-of-pocket health expenditure which brings in its wake catastrophic health expenditure and impoverishment could be partially explained by increasing unmet demands of patients attending public facilities as a result of inefficient use of resources and who in turn have recourse to health care privately against payment. The impact of catastrophic health expenditure and impoverishment due to out of pocket health expenditure is very likely to be exacerbated in the wake of COVID-19, unless public sector regional public hospitals improve its efficiency to service better the needs of patients. At present Mauritius does not have a national social health insurance scheme. Private Health expenditure is mainly in the form of out of pocket with only 6% related to premiums for private insurance [18]. The government announced a few years back its intent to introduce voluntary health insurance scheme for public employees, where the state will contribute 50% of premium in favour of civil servants, who are willing to seek medical care in the private sector. The introduction of this health insurance scheme targeting civil servants will ease public sector hospital services. Financial savings to be ensued would increase available resource envelope for hospital inputs and contribute to hospital efficiency and productivity [53].

### Limitations

The output variable assumes that all patients attending the outpatient departments or admitted are similar and there is no dichotomy in the quality of care provided. Patients Refined Diagnosis Related Groups (DRG) classification system with patients clustered according to their reason of admission, severity of illness and risk of mortality are not available in Mauritius. Thus, the study did not consider the aspect of case mix and severity of illnesses. The empirical model used in the study captures most of the inputs relevant to human resources through doctors, nurses and nonmedical staff but pertaining to capital inputs it is limited to beds. Considering only beds as a proxy for capitals inputs assumes that the level health technology is uniform across all the public hospitals. Disaggregated data in the panel at the level of each five regional hospital was available for the first 6 years only (2001–2006) while for the remaining 11 years only aggregated data for the five hospitals were considered for the hospital production function.

## Conclusion

This study asserts that public hospitals have relatively high technical efficiency score and increasing returns to scale. Notwithstanding that technical efficiency score ranges between 0.84 to 0.89, depending on the applicable function model, achieving full efficiency would allow for significant expansion in fiscal space. The potential fiscal space expansion, estimated at 8.9% of GGHE in fiscal year 2021–2022 and 9.2% of GGHE in fiscal year 2022–2023, can fund critical programmes such as HIV, vaccine preventable diseases, and responses to emerging infectious diseases. In the wake of COVID-19 outbreak and the challenges this would have on government revenues, improving efficiency and creation of fiscal space will be of essence for any future health financing strategies to be adopted.

Institutionalisation of technical hospital efficiency measurement as well as for health facilities at other levels of the health care delivery system is an important step for sound health policy making and health planning that needs to be prioritised. Informed decisions regarding which health facilities needs to be either scaled up or down and determining the scope of potential savings to create fiscal space can be made based on technical efficiency analysis. In the same breath, analysing hospital efficiency on a case mix basis and according to severity of illness as well as developing an All Patient Refined DRG in Mauritius will allow comparison of performance across hospitals. Moreover, it will have a positive impact on improving efficiency as already experienced in other countries.

## Data Availability

Please contact author for data requests.

## Abbreviations

COVID-19: Coronavirus disease 2019
DEA: Data Envelopment Analysis
DRG: Diagnostic Related Groups
GDP: Gross Domestic Product
GGHE: General Government Health Expenditure
MoHW: Ministry of Health and Wellness
PHC: Primary Health Care
PPF: Production Possibility Frontier
SFA: Stochastic Frontier Analysis
UHC: Universal Health Coverage
WHO: World Health Organization
